# Effects of a rehabilitative whole-body resistance band wrap on equine gait, posture, cortisol, and muscular function

**DOI:** 10.3389/fvets.2025.1738766

**Published:** 2026-01-28

**Authors:** Brooke Boger, Maegha Naraian, Emily Hernandez, Alexis Eaton, Ruby Rockburn, Isabella Tillman, Stesha Payne, Chelsey Yob, Char Panek, Jane M. Manfredi

**Affiliations:** 1 McPhail Equine Performance Center, College of Veterinary Medicine, Michigan State University, East Lansing, MI, United States; 2 Department of Large Animal Clinical Sciences, College of Veterinary Medicine, Michigan State University, East Lansing, MI, United States; 3 Department of Pathobiology and Diagnostic Investigation, College of Veterinary Medicine, Michigan State University, East Lansing, MI, United States

**Keywords:** acoustic myography, horse, lameness, rehabilitation, sports medicine, stress

## Abstract

Resistance bands used while horses are exercised with their handlers have shown benefits, but it is unknown if whole-body resistance bands used independently have therapeutic benefits. This study hypothesized that horses with varying gait asymmetries would experience improvements in lameness, muscular function, range of motion, posture, and cortisol following short-term use of a whole-body resistance band wrap (RBW). In this study, nine lame adult horses were evaluated with and without the RBW. The assessment included: objective gait analysis, acoustic myography, postural analysis, gait kinematics, and salivary cortisol concentrations. Statistical analyses included: Shapiro–Wilk Test, Paired Student T-Tests or non-parametric Wilcoxon Signed-Rank Tests (significant at *p* < 0.05). There was no difference in lameness, velocity, or stride length. Cortisol levels were lower while wearing the RBW (*p* = 0.03). The RBW decreased semitendinosus muscle efficiency (EST Score, *p* = 0.008), and increased carpal (*p* = 0.03), tarsal (*p* = 0.03), and shoulder (*p* = 0.03) joint range of motion (ROM). Back angle increased when wearing the RBW (*p* = 0.04). These findings indicate the RBW has short-term effects on decreasing cortisol, improving joint ROM, and decreasing semitendinosus muscle function. Future studies exploring the use of the RBW with different exercise protocols are needed to further clarify its use for equine rehabilitation.

## 1 Introduction

The etiology of lameness is multifaceted, involving a wide range of conditions affecting bones, joints, muscles, tendons, and ligaments, of which osteoarthritis is most common (1). Horses with lameness will adopt altered gait patterns to redistribute their weight and manage pain, which can result in a change in muscle activation patterns (2, 3). Management of lameness often requires a multimodal approach, including systemic medication, intra-articular therapies, and rehabilitation of compensatory changes (4). It is essential to provide treatment for the inciting cause of equine lameness. However, coupled with rehabilitative assistance, both non-athletic and athletic horses can restore their normal functional capacity and athletic potential (5).

A review published in 2021 found that exercise, electrotherapy, and hydrotherapy were the most commonly used rehabilitative techniques in horses (5). Additionally, in a survey conducted in 2018, controlled hand walking comprised 97.3% of the exercise completed across eight veterinary groups (6). This reliance on controlled hand walking alone highlights the need to evaluate rehabilitative methods that can be used while hand walking that can address the compensatory changes brought on by lameness and improve range of motion, proprioception, and strength.

In humans, resistance bands have been shown to reduce pain, enhance proprioception, and improve muscle strength and function in women with knee OA (7). In horses, a popular equine resistance band system (Equiband® System) has been found to increase dynamic stability of the thoracolumbar region of lame and non-lame horses and increase hind end symmetry in lame horses after 4 weeks of use (8, 9). Additionally, this resistance band system has been evaluated for its effect on core muscle activation, and it was found that muscle activation of the rectus abdominus increased when horses wore the system (10). This resistance band system does not fully wrap around the horse, offering no proprioceptive input of the front end. Consequently, it requires a handler to either work the horse in hand or ride them to obtain the rehabilitative benefits. This highlights the need for research into other resistance band options such as a whole-body resistance band wrap (RBW) that the horse can wear without needing a handler present.

The Eagle ProSix equine system, a RBW, is believed to improve proprioception, gait symmetry, posture, and muscle function in horses. Additionally, this system applies deep touch pressure around the entire horse and is thought to help relieve stress and anxiety. The objectives of this study were to investigate the short-term effects of applying the RBW on several parameters relating to stress, gait, and muscular activity. This study hypothesized that horses with varying gait asymmetries would experience improvements in lameness, muscular function, range of motion, posture, and cortisol while wearing the RBW following short-term use.

## 2 Materials and methods

### 2.1 Animals

Nine horses were used from our institutions teaching herd. All horses included in the study had pre-existing gait asymmetries (AAEP grade of 3/5 in one or more limbs). Based on previously published work on resistance bands in horses, six horses were deemed necessary to achieve significance (8–10). This was supported by a power analysis (G*Power 3.1.9.2) looking at both an objective forelimb lameness mean difference of 8 and standard deviation of 5 and an AMG EST score for the rectus abdominus mean difference of 5 with a standard deviation of 3, both with a power of 80% and an alpha of 0.05. Nine horses were chosen in case of unforeseen circumstances (8–10). This study was approved by our institution’s Institutional Animal Care and Use Committee (IACUC) PROTO202400094.

### 2.2 RBW adjustment

Each horse was sized and fitted with a RBW that was then kept for only that horse for the length of the study. The horses were weighed and then measured around their abdominal circumference (widest part of the abdomen) and body circumference (as measured at the level of the point of the shoulder around the whole body while staying level to the ground) as directed by the company’s sizing guide to determine which size they would fit in (small, medium or medium-large were used for the horses in this study). The wrap was positioned to allow the band around the hind-end to sit comfortably just under the tuber ischium and the front-end of the band to sit on the chest below where the neck meets the chest. The RBW’s hip tensioners were tightened to just below the amount which would cause the band to slip down in the back while walking in order to standardize its fit between horses of different body shapes. The first band over the back was adjusted to sit just in front of the withers and the last strap was adjusted to sit in front of the tuber sacrale. The abdominal band was pulled snug over the middle of the abdomen.

### 2.3 Short-term RBW application

Horses were randomly assigned to either wear (*n* = 4) or not wear (*n* = 5) the RBW (Eagle ProSix, Bark River, MI) first. After a 15-min acclimation period while walking, lameness evaluation, gait analysis, posture analysis, and acoustic myography were performed for each condition (horses assigned to wear the RBW were assessed in the RBW). The conditions were then switched, and after 1–4 h, an additional 15-min acclimation period was observed, followed by the same assessments.

### 2.4 Objective lameness evaluation

For the objective lameness evaluation, a body-mounted inertial sensor system was used (Lameness Locator, Equinosis Q, Columbia, MO) (11). This system uses sensors placed on the horse’s head, right forelimb pastern, and pelvis and is validated to report equine asymmetry in vertical head and pelvic positions (11). All horses were trotted by the same handler on the same hard ground on a straight line (approximately 30 meters).

### 2.5 Acoustic myography

**Figure 1:**
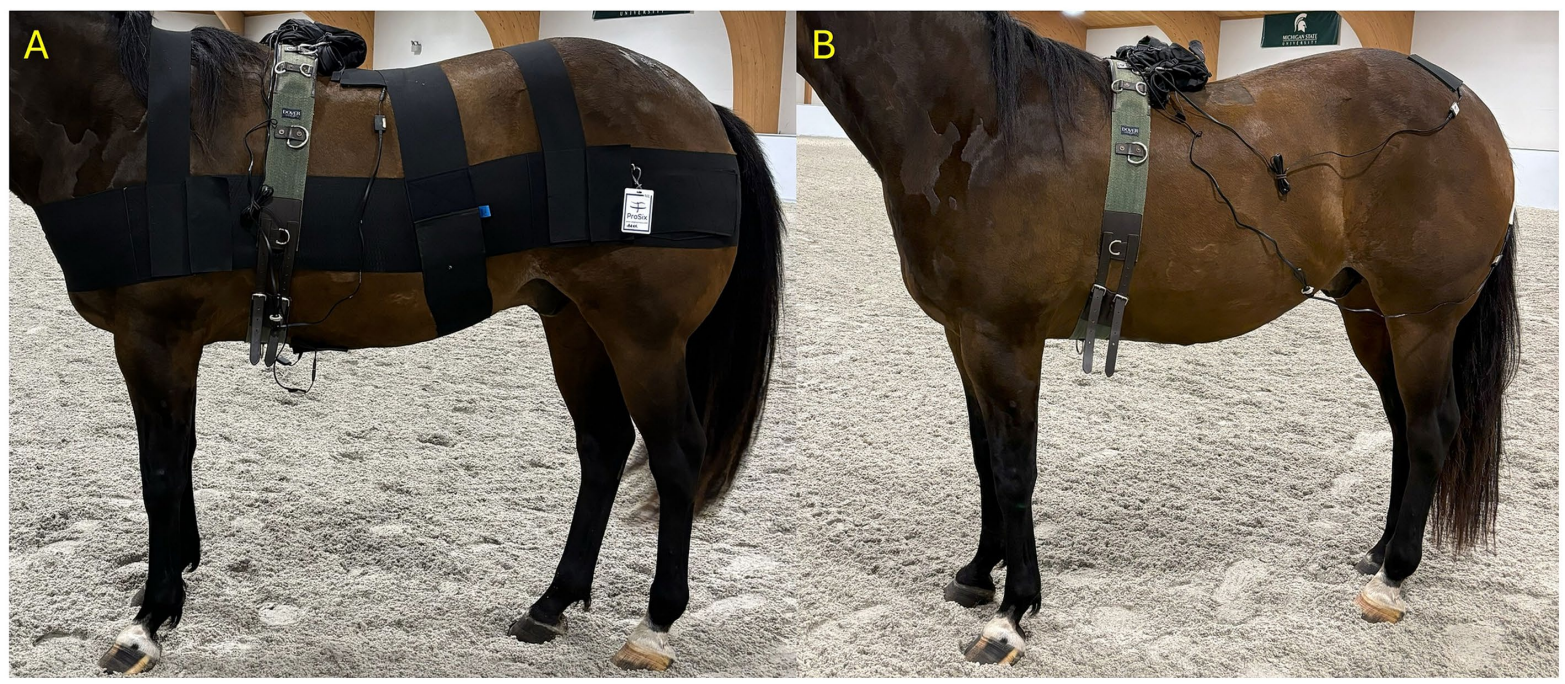
Placement of AMG sensors over the longissimus dorsi and rectus abdominis muscles **(A)** and over the gluteus medius and semitendinosus muscles **(B)**.

An acoustic myography (AMG) recording device (CURO Diagnostics ApS, Bagsavared, Denmark) was used to take recordings with and without RBW application (12, 13). The 20 mm sensors (MyoDynamik Sensors, Copenhagen, Denmark) were placed over the four muscle groups: gluteus medius, semitendinosus, longissimus dorsi, and rectus abdominis muscles at the same level on both the right and left side (Figure 1). The gluteus medius sensors were placed at the midpoint of the muscle at the widest point after identifying the tuber coxae, tuber sacrale, and the 3rd trochanter of the femur (14). The semitendinosus sensors were placed midway between the tuber ischii and the caudal aspect of the stifle (15). The longissimus dorsi sensors were placed approximately 2 cm lateral to midline at approximately the level of the dorsal spinous process T16 (10). The rectus abdominis sensor was placed 2 cm lateral to the ventral midline along the muscle belly and in front of the abdominal band of the RBW (10). Muscle groups were measured in pairs. The gluteus medius and semitendinosus muscles were measured at the same time with 4 different sensors (one each for left gluteus medius, right gluteus medius, left semitendinosis and right semitendinosis), and the left and right longissimus dorsi and rectus abdominis muscles were measured at the same time similarly to above. Three AMG recordings at the trot for each pair of muscles were performed in the same area and for the same distance where the lameness evaluation was performed. The mean of the three passes was used for data analysis.

For set up before taking the AMG measurements, a surcingle was placed on each horse and adjusted to a comfortable tightness prior to placing the sensors. Clipping of the hair coat was not necessary as it was summer, the horse’s coats were thin, and AMG sensors can work effectively with some hair present. Ultrasound gel was placed on the horse’s skin and the sensor for the best sound transmittance. The sensor was then placed on the desired muscle group and secured to the horse using an adhesive foam bandage (3 M, St. Paul MN). Each sensor was then attached to the AMG recording unit using a cable, and the unit was adhered to the horse by being placed in a small backpack attached to the surcingle (Figure 1). For each recording, the “record” button was used on the AMG recording device and the AMG signal was transmitted to a hand-held tablet (iPad, Apple Inc., Cupertino, CA) to view in real-time to confirm the signal was appropriate and that contact was maintained with the sensors.

The recordings were analyzed using the Curo Analysis Website (Myodynamik, Bagsavared, Denmark). The maximum frequency (max T) was 160 Hz, and the threshold was 0.2 (16). If any one of the E-, S-, or T-scores showed up as 0 or 10 (maximum values), the threshold was then adjusted down to 0 and increased until the first incidence occurred of all E-, S-, and T-scores displaying a non-zero number. The resulting values give the amount of time a muscle is contracting or resting (E-score), the spatial summation, which is the number of contracting fibers (S-score), and the frequency of contractions (T-score) for the left and right side of each muscle group (17). For each muscle group, the mean E, S, and T-score, as well as the EST score were calculated.

### 2.6 Gait kinematics

Video recordings using a portable media device (iPhone 12Pro, Apple, CA, set to record at 60 fps) were taken of each horse trotting at their desired speed by hand, with and without the RBW. Line calibration was obtained by videoing a calibration yard stick in the path the horses traveled on. In the program a line object was placed on top of the ward stick and the real-world length entered. The horses were trotted at their preferred speed over the same hard-packed 20-meter-long path with the same handler for every recording. The recording device was placed on a tripod at the level of the point of the shoulder 9.7 meters away from the center of the path where horses would be trotting. Each horse had two video recordings taken to display both their right and left sides for each condition. Gait analysis was performed using kinematics software (Kinovea v.0.9.5). Four stride lengths (SL) were calculated from each video, with the definition of a SL being the initial point of contact of the front foot nearest the camera to the next point of contact after the limb completed one swing phase (18). The SLs were then averaged to give an overall mean SL for each horse under each condition. Velocity was determined by calculating the mean of four time points from each video, using the time taken to cover a given distance to obtain an overall mean velocity under each condition.

For analyzing joint angles, approximately 2.54 × 2.54 cm reflective markers were placed on the horses using white tape. On the thoracic limb, markers were placed on the lateral hoof wall, lateral metacarpal epicondyle, lateral styloid process, lateral humeral epicondyle, and the greater tubercle of the humerus (19). And for the pelvic limb, markers were placed on the lateral hoof wall, lateral metatarsal epicondyle, lateral tubercle of the talus, lateral femoral epicondyle, and the greater trochanter of the femur (20). Angles of the carpus, tarsus, and shoulder were evaluated for this study. The angular joint range of motion (ROM) was defined as the difference between the maximum and minimum joint angle achieved by a joint in a stride (21). For each horse, the mean of four ROMs from each side was calculated, giving an overall mean ROM under each condition.

### 2.7 Posture

**Figure 2:**
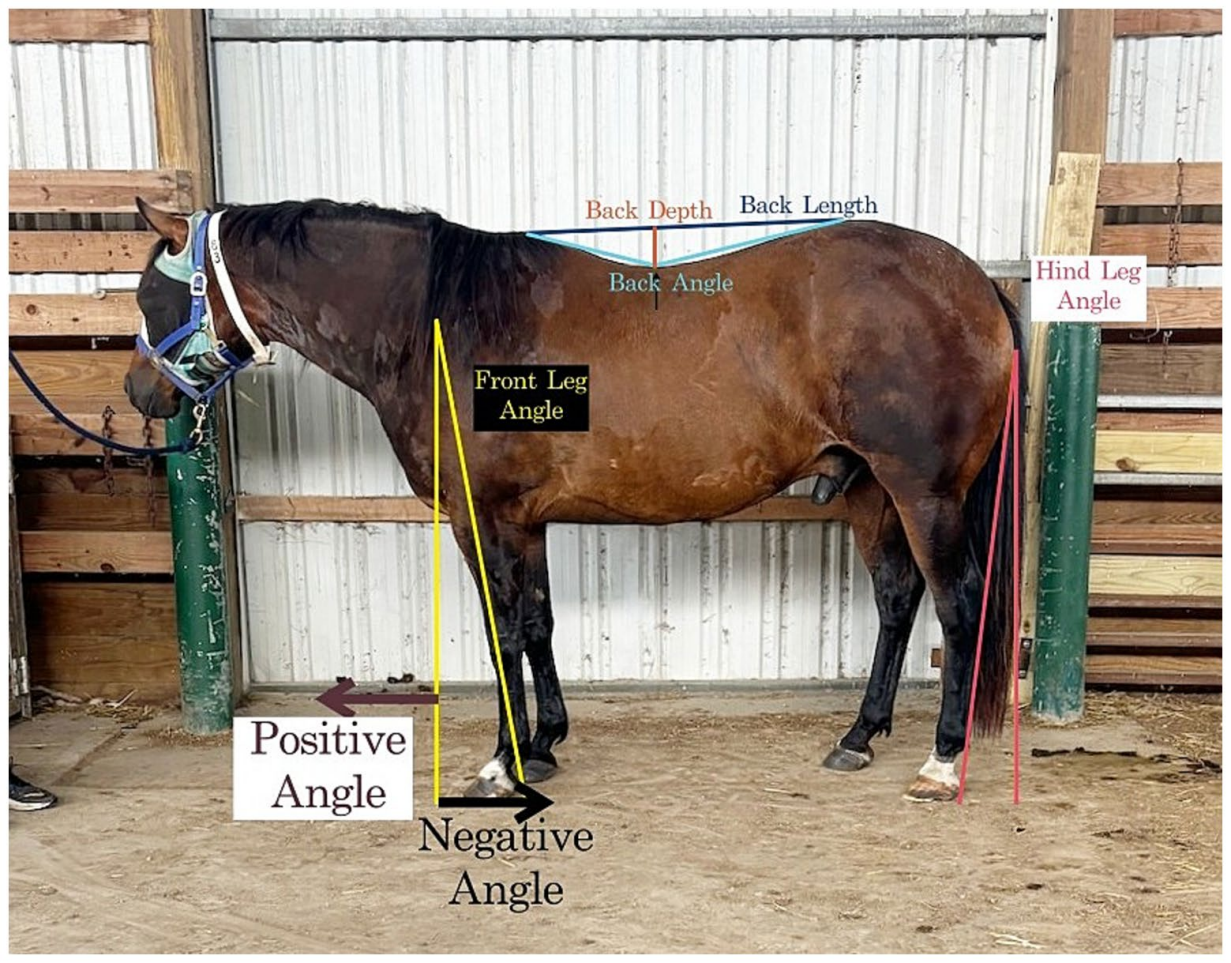
Explanatory picture for each of the postural components measured with and without the whole-body resistance band wrap (RBW).

Pictures were taken of each horse with and without the RBW. For taking the photos, the horses were walked to a standard position and halted, allowing them to maintain their chosen stance and head position. This was repeated to obtain both left and the right lateral photographs of each horse both with and without the RBW. Measurements obtained from both the right and left images were then averaged for each condition. Pictures were taken using an iPhone camera (iPhone 12 Pro, Apple, CA) from a distance of 5.5 meters. Posture components were analyzed using imaging processing software (Image J v.1.53). Images were processed to add lines that indicate the length of the horses’ back, the lowest point of the horses’ back, the highest point of the wither, and the highest point of the back at the tuber sacrale (PhotoScape X v.4.2.1). A straight line was added from the ground to the middle of the spine of the horse’s scapula, which was then extended to go through the middle of the horse’s fetlock to show the angle of the front leg placement (22). In addition, a straight line was added from the ground to the point of the buttock, which was extended to follow the hind leg’s superficial digital flexor tendon (22). The same set measurements were used in all images to keep consistency. The measurement of the limb angles was based on the vertical line (22). If the animal had its limb caudally to the drawn line, then the angle was recorded as negative. If the animal had its limb cranially to the line, then the angle was recorded as positive. The set measurement was the width of the door behind the horses, which was 292 cm. The back area measurement was done by following the horse’s back conformation from the highest point of the wither to the highest point of the buttock (23). For the back angle, the measurement was taken from the highest point of the wither to the lowest point of the back to the highest point of the buttock (23). Back length was measured as a straight line from the highest point of the wither to the highest point of the buttock. The back depth was measured from the lowest point of the back to the height that intersected the back length line. Horses were said to be standing square in the front if the front limbs overlapped with each other so that only one leg was visible. The same is true for hind limb squareness. Figure 2 describes each of the postural components measured. The same author performed all these measurements.

### 2.8 Cortisol

Cortisol was measured at the same time each day between the hours of six thirty and eight AM. Horses were brought into a stall that they were familiar with the day before starting cortisol measurements and remained in the stall until the completion of day two. The horses were fasted from 10 p.m. the night before with only access to water (horses were used to this feeding regimen). On the first day, half of the horses were randomly assigned to wear the RBW, while the other half did not. On the second day, the conditions were switched. Cotton swabs (Salimetrics LLC, Carlsbad, CA) were used to collect saliva at 0 min and 45 min after wearing the wrap or after baseline collection for those not wearing the wrap. For saliva collection, cotton swabs were held by the handlers and swiped over the tongue and on the inside of the cheek, allowing the horse to wet the cotton for 30–45 s; this was repeated four times for each collection point. The swabs were each placed in a centrifuge tube (Salimetrics LLC, Carlsbad, CA) and were centrifuged at 1500 x g for 5 min. Saliva was pipetted from the bottom of the centrifuge tube into microcentrifuge tubes, and all samples were frozen at −80 °C for at least 3 h prior to analysis. Cortisol concentrations were analyzed using a salivary cortisol-specific ELISA validated for equine samples (Salimetrics LLC, Carlsbad, CA) following all manufacturer instructions (24). The intra-assay coefficient of variation (CV%) was 6.3%. Only six of the horses were able to participate in the cortisol portion of this study due to a limited number of stalls and horse injuries unrelated to the study that occurred after all of the previous lameness, gait, and muscle measurements were obtained but before the planned day for cortisol measurement.

### 2.9 Statistics

Dedicated statistical software (Graph Pad Prism 9, La Jolla, CA) was used to assess short -term effects on gait kinematics, posture, muscle function, and cortisol. A Shapiro–Wilk Test was used to assess normality. Paired Student T-Tests or non-parametric Wilcoxon Signed-Rank Tests were used to compare short-term application effects on lameness, muscle function, posture, stride length, speed, joint angles, and cortisol concentrations pre- and post-wearing the RBW. Significance was set at *p* < 0.05.

## 3 Results

### 3.1 Animals

The nine horses had ages ranging from 7 to 23 years old (mean 17 years, standard deviation 5 years). The breeds included four Quarter Horses, two Standardbreds, one Haflinger, one Draft Cross, and one Appaloosa. Musculoskeletal conditions included: a fetlock osteochondral fragment (*n* = 1) and arthritis of the coffin (*n* = 2), carpus (*n* = 2), fetlock (*n* = 2) and/or tarsal joints (*n* = 5). There was one gelding and the rest were mares.

### 3.2 Objective lameness

**Figure 3:**
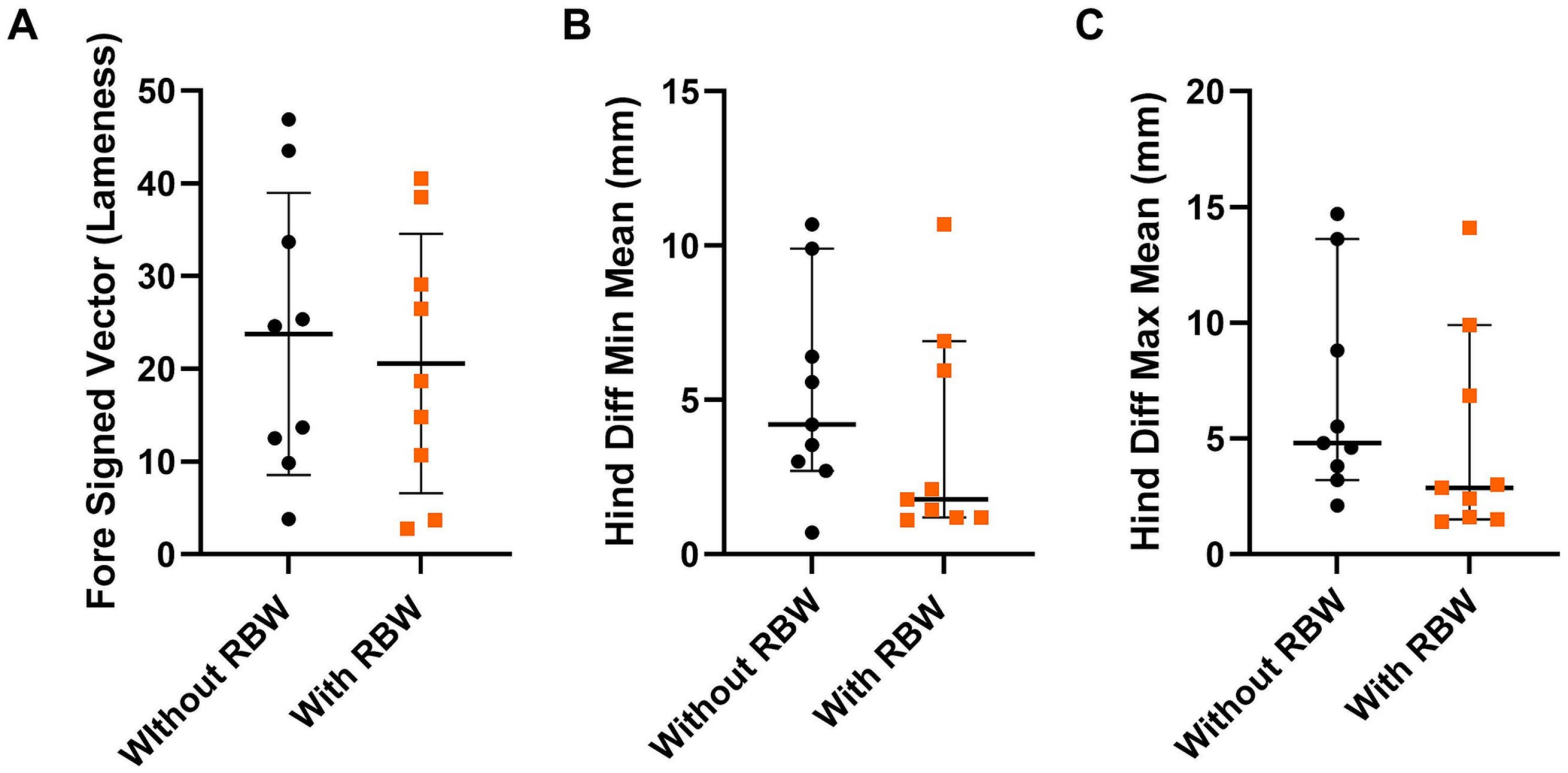
Mean ± SD of fore signed vector **(A)** and median ± 95% CI of hind diff min mean **(B)**, and hind diff max mean **(C)** with (orange squares) and without (black circles) the short-term application of the whole-body resistance band wrap (RBW; *n* = 9).

For short-term application of the RBW, there was no difference in forelimb signed vector sum (*p* = 0.4), hind diff min mean (*p* = 0.25), or hind diff max mean (*p* = 0.13) with or without the RBW (Figure 3). The mean ± SD pre-RBW forelimb signed vector was 33 ± 19, the hind diff mean was 6 ± 6 and the hind diff max was 5 ± 6.

### 3.3 Acoustic myography

**Figure 4:**
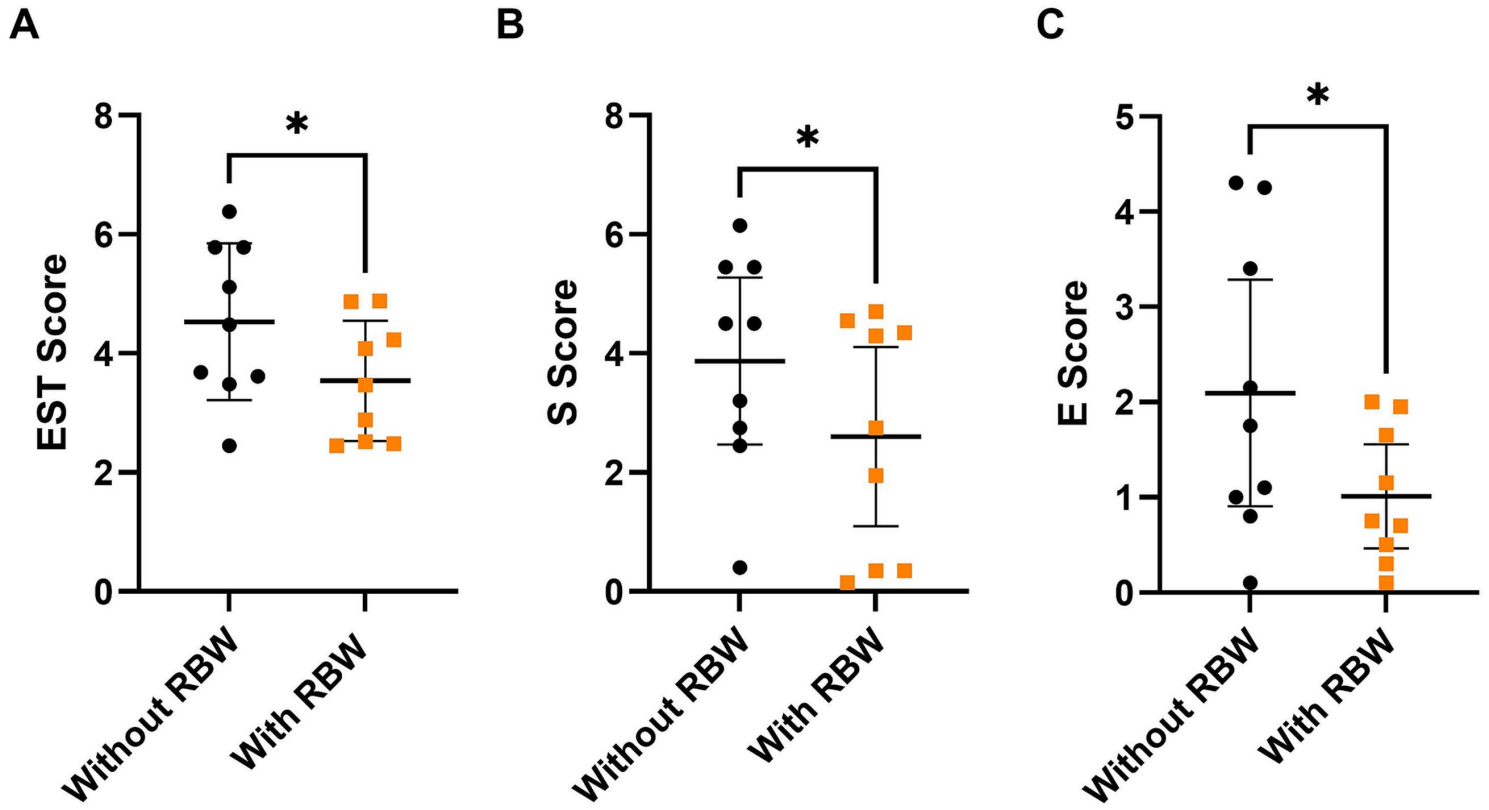
Mean ± SD of semitendinosus EST score **(A)** and median ± 95% CI of S score **(B)**, and E score **(C)** with (orange squares) and without (black circles) the short-term application of the whole-body resistance band wrap (RBW; *n* = 9). *p < 0.05.

The mean ± standard deviation of each score for each of the muscles can be found in Table 1. There was no difference in E, S, T or EST score of the gluteus medius, longissimus dorsi, or rectus abdominis muscles (all *p* > 0.05) with or without the RBW. However, for the semitendinosus muscle, there was a significant decrease in E score (*p* = 0.02), T score (*p* = 0.02), and EST score (*p* = 0.008) after wearing the RBW, but not S score (*p* = 0.07), indicating a decrease in semitendinosus muscle activity while wearing the RBW (Figure 4).

**Table 1:** Mean ± standard deviation of each of the acoustic myography value for each muscle group with and without the whole-body resistance band wrap (RBW) averaged over all of the horses for that condition (*N* = 9).

Acoustic myography measurement	Without RBW	With RBW	*p* value
Gluteus Medius			
E score	1.9 ± 1.0	2.5 ± 1.6	0.6
S score	5.4 ± 1.2	5.5 ± 0.9	>0.9
T score	8.1 ± 1.5	8.2 ± 0.6	0.8
EST score	5.1 ± 0.8	5.4 ± 0.8	0.6
Semitendinosus			
E score	2.1 ± 1.5	1.0 ± 0.7	0.02
S score	3.9 ± 1.8	2.6 ± 2.0	0.07
T score	7.6 ± 1.4	7.0 ± 1.3	0.02
EST score	4.5 ± 1.3	3.5 ± 1.0	0.008
Longissimus dorsi			
E score	3.5 ± 2.6	4.0 ± 1.8	0.6
S score	6.3 ± 2.2	7.2 ± 0.8	0.9
T score	7.4 ± 1.6	7.9 ± 1.6	0.3
EST score	5.7 ± 1.8	6.4 ± 1.2	0.8
Rectus abdominis			
E score	5.0 ± 2.7	5.2 ± 2.4	0.8
S score	7.6 ± 0.5	7.3 ± 0.8	0.7
T score	7.8 ± 1.4	7.8 ± 1.5	0.9
EST score	6.8 ± 1.2	6.8 ± 1.3	0.9

### 3.4 Stride length and velocity

The mean ± SD of SL without the RBW was 2.2 ± 0.2 meters, and with the RBW was 2.3 ± 0.1 meters. There was no difference in mean SL for the short-term application of the RBW (*p* = 0.45). The mean ± SD of velocity without the RBW was 3.5 ± 0.6 m/s, and with the RBW it was 3.5 ± 0.3 m/s. There was no difference in mean velocity for the short-term application of the RBW (*p* = 0.97).

### 3.5 Joint range of motion

**Figure 5:**
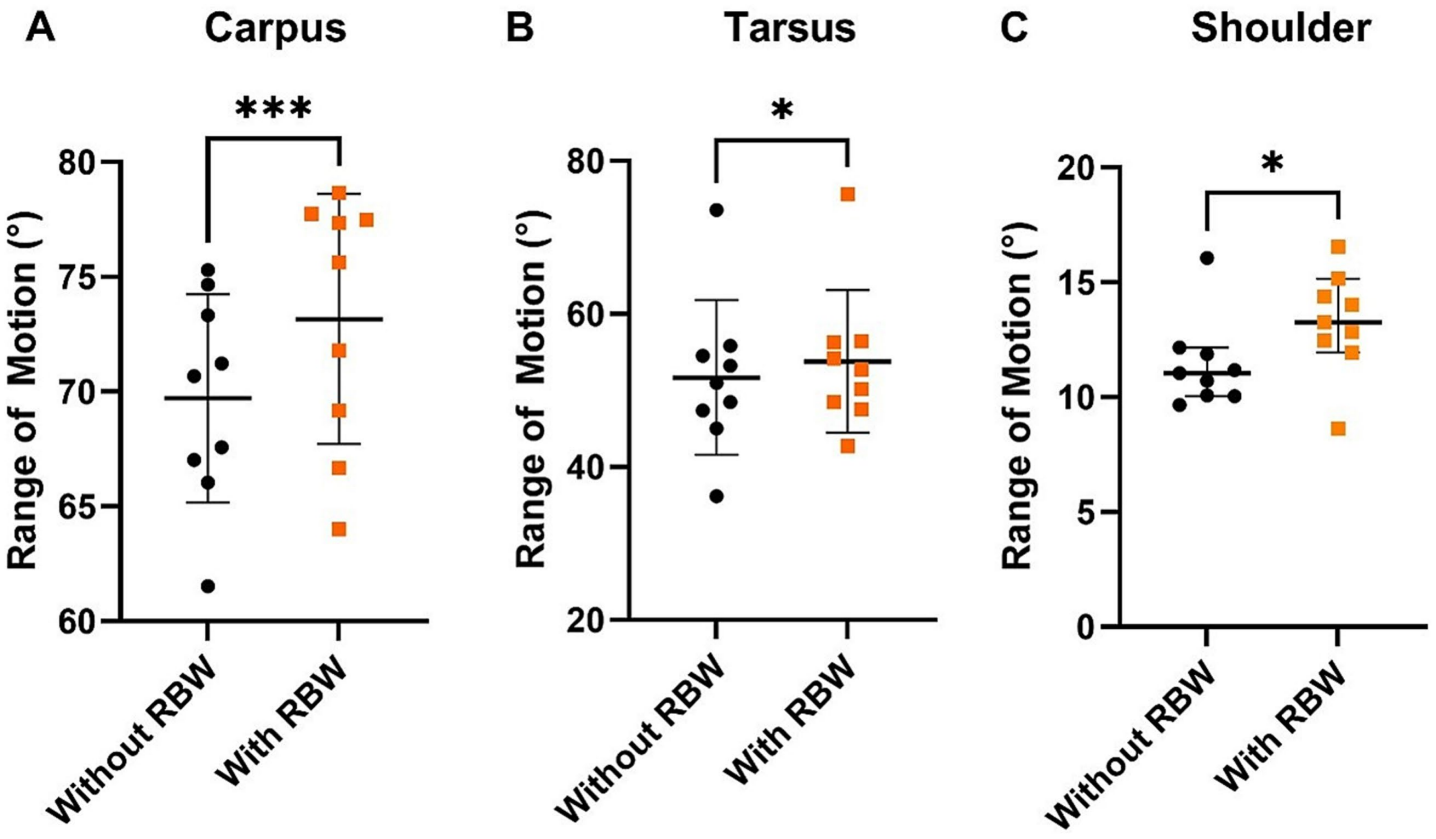
Mean ± SD of carpus range of motion (ROM) **(A)** and tarsus ROM **(B)** and median ± 95% CI of shoulder ROM **(C)** with (orange squares) and without (black circles) the short-term application of the whole-body resistance band wrap (RBW; *n* = 9). *p < 0.05, *** = *p* < 0.001.

For the short-term application of the RBW, the carpus ROM increased from 69 ± 4° to 73 ± 5° (Figure 5) when wearing the RBW (*p* = 0.03).

For the short-term application of the RBW, the tarsus ROM increased from 51 ± 10° to 53 ± 9.° (Figure 5) when wearing the RBW(*p* = 0.03).

For short-term application of the RBW, the shoulder ROM increased from 11 ± 2° to 13 ± 8° (Figure 5) when wearing the RBW (*p* = 0.03).

### 3.6 Posture analysis

**Figure 6:**
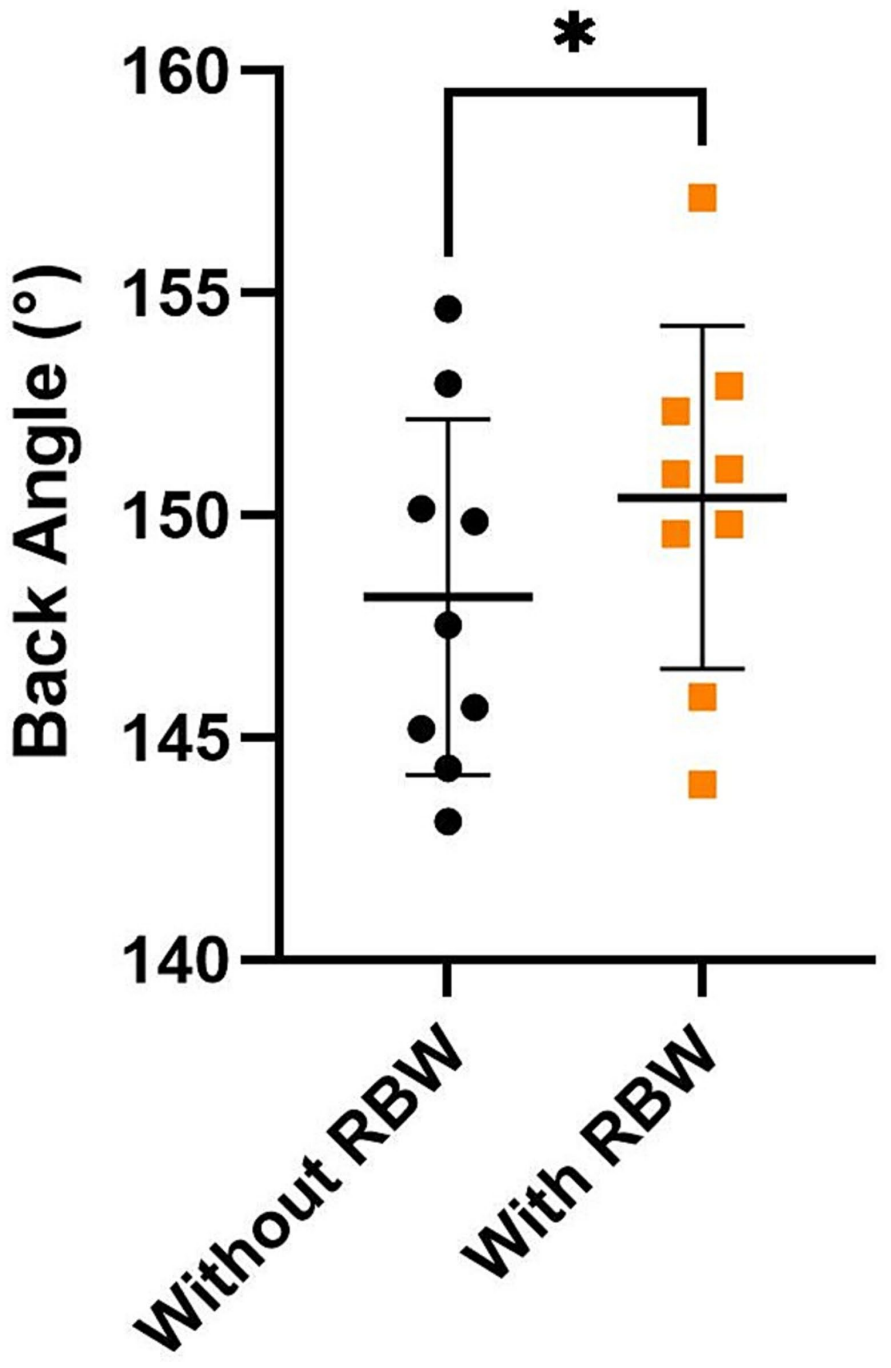
Mean ± SD of back angle with (orange squares) and without (black circles) the short-term application of the whole-body resistance band wrap (RBW; *n* = 9). *p < 0.05.

All postural components are displayed in Table 2. For changes in posture after short-term application of the RBW, there were no significant effects on the back area, back depth, back length, front leg angle, or hind leg angle (all *p* > 0.05). However, there was a significant increase in back angle after short-term application of the RBW (*p* = 0.04), where mean back angle increased from 148.2 ± 4.0° to 150.4 ± 3.8° (Figure 6).

**Table 2:** Mean ± standard deviation of each posture component measured with and without the whole-body resistance band wrap (RBW) averaged over all of the horses for that condition (*N* = 9).

Measurements	Without RBW	With RBW	*p* value
Back area (cm)	699.2 ± 60.1	731.0 ± 106.1	0.06
Back depth (cm)	12.5 ± 1.0	12.8 ± 1.5	0.5
Back length (cm)	96.1 ± 4.2	98.8 ± 5.9	0.05
Back angle (degrees)	148.2 ± 4.0	150.4 ± 3.9	0.04
Front leg angle (degrees)	−9.8 ± 3.0	−7.3 ± 4.4	0.15
Hind leg angle (degrees)	2.0 ± 7.5	7.6 ± 4.2	0.09

### 3.7 Cortisol

**Figure 7:**
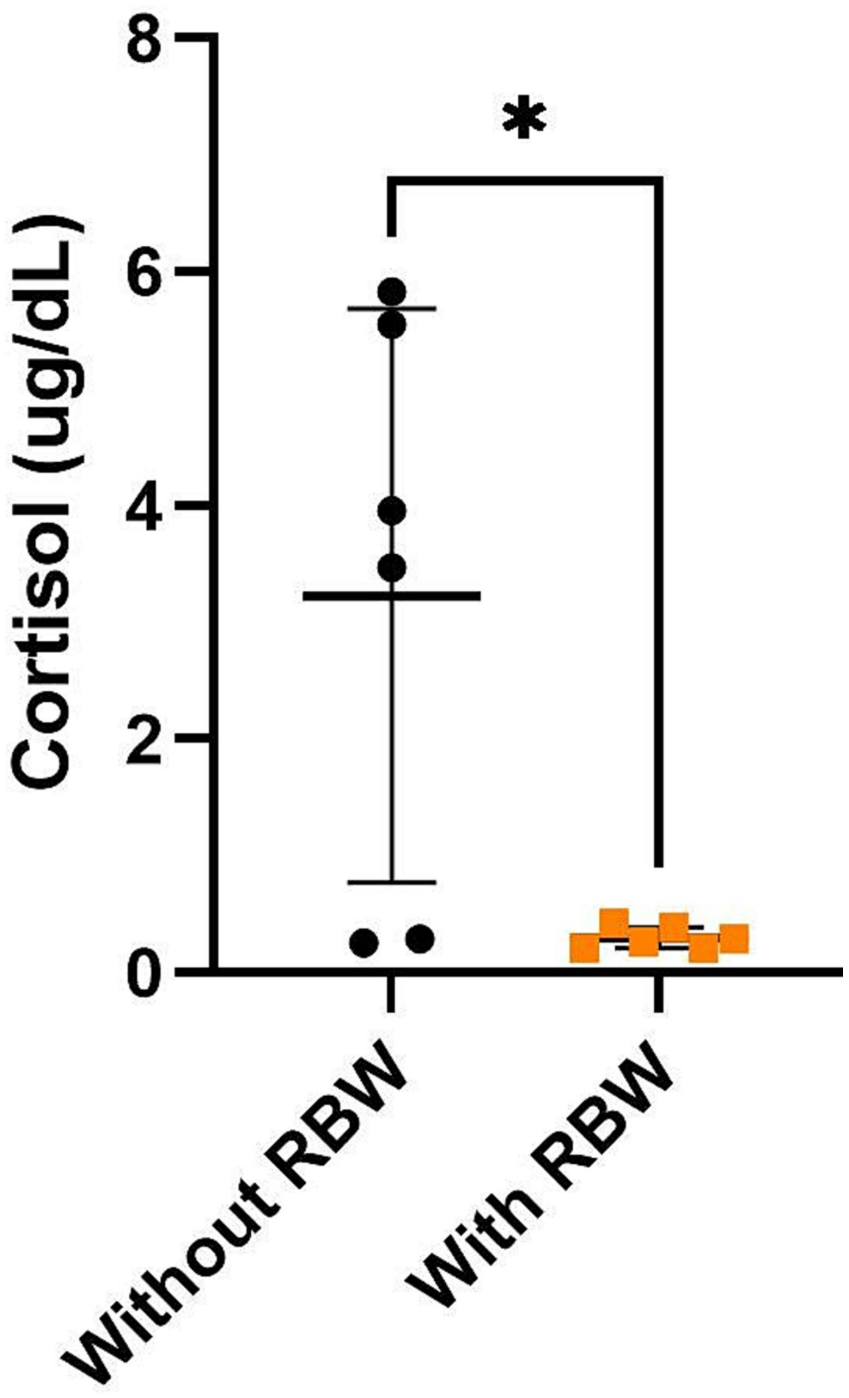
Mean ± SD of cortisol (μg/dL) 45 min after baseline with (orange squares) and without (black circles) the whole-body resistance band wrap (RBW; *n* = 6). *p < 0.05.

Horses wearing the RBW had lower cortisol concentrations than those not wearing the RBW at the 45 min time point (Figure 7; *p* = 0.03).

## 4 Discussion

This study found that the short-term application of the RBW resulted in increased ROM in the carpus, tarsus, and shoulder, as well as an increased back angle, and a decrease in both semitendinosus muscle function and cortisol concentration. This study did not find any changes with lameness after short-term use of the RBW. Therefore, the hypothesis that horses with varying gait asymmetries would experience improvements in lameness, muscular function, range of motion, posture, and cortisol while wearing the RBW following short-term use was only partially accepted.

The results of this study were similar to those of previous research on resistance bands in horses, which found no improvement in lameness after short-term application (8). Due to the lack of improvement in lameness, it remains necessary to address the primary cause of lameness in horses by other means, with use of the RBW as an adjunctive rehabilitation method.

This study found altered semitendinosus muscle function while wearing the RBW, which could be due to muscle fatigue or decreased use resulting from the band directly wrapping around these muscles. All the other muscles did not demonstrate a change in function with or without the RBW. Because of this finding, horses looking to increase muscular activity in the semitendinosis muscle (e.g., a race horse or eventer after a long lay up after a hind limb injury) should seek other interventions, and continuous use of the RBW should not be pursued in a rehabilitation program. A previous equine study found that wearing resistance bands increases muscle activation in the rectus abdominis muscle but reduces muscle activation in the longissimus dorsi muscle, suggesting that resistance bands can alter each muscle’s function differently (10). Our study did not observe these changes in the rectus abdominis or longissimus dorsi; however, this could be attributed to the differences in the type of resistance bands used. Additionally, this current study utilized AMG to measure muscle activity, whereas the previous equine study employed surface electromyography (sEMG); therefore, the difference in techniques could have yielded different results. sEMG is more sensitive to nearby electronic equipment and signals may be affected by nearby muscle’s motor unit action potentials.

Elastic resistance bands placed around a horse’s hindquarters are a common therapeutic technique thought to induce hindquarter protraction (25). This study found short-term improvements in range of motion of the carpus, tarsus, and shoulder joint while wearing the RBW. While no current studies have evaluated joint ROM while wearing resistance bands in humans or horses, these results agree with previous research that found kinesiology tape, which provides proprioceptive input like the RBW, increases joint ROM in humans (26). Based on these findings, the RBW may be useful in aiding the rehabilitation of carpal, tarsal, and shoulder injuries. There were no differences found for velocity or stride length when evaluating the short-term effects of the RBW. This agrees with a previous study that found no difference in stride time when wearing elastic resistance bands (9), and indicates that the RBW does not hinder the forward movement of the horse while in the trot.

Posture was evaluated in this study as it can indicate the animal’s balance and potential pain (27). Underlying issues, such as lameness, can lead to changes in posture to help compensate for lingering pain, and the use of non-invasive therapy methods, like the RBW, may help correct these changes (28). The repeated use of non-invasive therapy methods helps the animal to exercise in a way that encourages correct muscle engagement (10). In this study, the back angle was found to increase while wearing the RBW. This indicates that while wearing the RBW, horses had improved topline posture, potentially by engaging the hypaxial musculature that AMG is unable to assess. This finding aligns with previous research that has shown activation of some core muscles when wearing elastic resistance bands during trotting (10). Horses having a more neutral (considered a good posture) versus a raised head position can also contribute to back shape changes. The changes in back posture observed in this study, which occurred while the animal remained static while wearing the RBW, indicate that the RBW may be used as a non-invasive method of rehabilitation to help with posture; however, longer term studies of its use to assess the possibility of developing habituation should be pursued.

In animals, moderate to deep pressure has been used as an alternative treatment for reducing tension and anxiety (29, 30). Deep pressure will influence both the parasympathetic and sympathetic nervous systems, leading to a decrease in the activation of the stress response (31). In this study, salivary cortisol levels were found to decrease when wearing the RBW compared to not wearing it. This finding aligns with previous research in humans, which has shown that wearing a therapeutic body wrap can decrease salivary cortisol levels (32). The RBW is designed to provide a whole-body pressure squeeze intended to aid in proprioception and stress relief. From these results, the RBW could be a useful rehabilitation tool for reducing stress in horses undergoing rehabilitation.

In conclusion, although the RBW did not demonstrate short-term improvements in lameness, it did improve joint range of motion and some indices of thoracolumbar posture while the horses were wearing it. This suggests that the RBW could be a useful rehabilitation tool for thoracolumbar dysfunction, especially in conjunction with treatments for the primary cause of lameness. Additionally, the RBW could be beneficial in helping to reduce stress in horses undergoing rehabilitation. This could be especially useful for horses on stall rest.

Limitations of this study include varied gait asymmetries, varying breeds and body types, use of a phone camera and not a dedicated motion capture system, and low enrollment numbers which could have led to an inability to see a significant improvement in lameness. While the horses had various degrees and types of osteoarthritis, we did feel it reflected what occurred in the general population of horses. However, post-hoc analysis of the fore- and hind limb lameness objective measures for these horses suggested that we would have needed closer to 40 horses to detect a significant improvement in lameness. Use of a single phone camera as compared to a dedicated motion capture system has limitations in sensitivity; however, it enables research to be done in the field. A previous study looking at dogs compared joint angles obtained from a phone/Kinovea system as compared to a dedicated motion capture system and found only a 2 degree difference between the two (33). In future, a similar study should be performed with horses, although Kinovea has been reported to be used for assessing horse kinematics previously (19).

Future research should focus on assessing how the RBW could be incorporated into pre-habiliation and rehabilitation exercise programs, as well as on how the RBW works in conjunction with traditional treatments for lameness or decreased range of joint motion, such as after the use of limb splints or casts, as well as with other conditions where muscle wasting is apparent such as with pituitary pars intermedia dysfunction or post colic surgery.

## Data Availability

The raw data supporting the conclusions of this article will be made available by the authors, without undue reservation.
